# Gerontologisches Gutachten zu fachlich begründeten Einzelleistungen nach § 71 SGB XII

**DOI:** 10.1007/s00391-024-02348-7

**Published:** 2024-09-19

**Authors:** Stefanie Engler, Christian Bleck, Cornelia Kricheldorff

**Affiliations:** 1 Evangelische Hochschule Freiburg, Bugginger Straße 38, 79114 Freiburg, Deutschland; 2 Hochschule Düsseldorf, Düsseldorf, Deutschland; 3 Katholische Hochschule Freiburg, Freiburg, Deutschland

**Keywords:** Altenhilfe, Gesetzgebung, Diversität, Gleichheit, Inklusion, Prekäre Lebenslagen im Alter, Soziale Arbeit, Care of old people, Legislation, Diversity, equality, inclusion, Precarious life situations in old age, Social work

## Abstract

**Hintergrund:**

Die Senatsverwaltung für Wissenschaft, Gesundheit und Pflege des Landes Berlin hat im 2. Halbjahr 2023 ein explizit gerontologisches Gutachten beim Autor*innen-Team in Auftrag gegeben; dieses sollte Anregungen zu Modernisierung und Konkretisierung des § 71 SGB XII, der zentralen sozialrechtlichen Grundlage für die sog. Altenhilfe, liefern. Es wurde zum Jahresende 2023 fertiggestellt. Die Zielsetzung war, über die Identifizierung, Analyse und Beschreibung von Einzelleistungen fachlich fundierte Anregungen zu formulieren für ein Berliner Altenhilfestrukturgesetz, das der Differenziertheit und Diversität der Lebensphase Alter Rechnung trägt.

**Ziel des Beitrags:**

Vorgehensweise und zentrale Ergebnisse des Gutachtens werden vorgestellt und diskutiert.

**Material und Methoden:**

In einem mehrschrittigen Vorgehen, verbunden mit einem multimodalen Forschungsansatz, wurden verschiedene Methoden miteinander verknüpft, um ein umfassendes Verständnis der aktuellen Situation der sog. Altenhilfe und gerontologische Begründungen ihrer Leistungen zu generieren. Gearbeitet wurde mit kritischen und selektiven Literaturreviews, Dokumentenanalysen und qualitativ-leitfadengestützten Expert*innen-Interviews.

**Ergebnisse und Diskussion:**

Das Gutachten enthält konkrete Empfehlungen zu altersphasen- und lebenslagenorientierten Entwicklung von Einzelleistungen nach § 71 SGB XII, die sich zum einen auf Zugänge, Formen und Themen der Beratung, die ein gelingendes Altern für alle alten Menschen ermöglichen und unterstützen, beziehen. Benannt werden zum anderen explizit notwendige und im methodischen Vorgehen identifizierte Geld- und Sachleistungen, die auch in prekären Lebenslagen im Alter Fähigkeiten zur Selbsthilfe stärken und Betroffenen ermöglichen sollen, selbstbestimmt am Leben in der Gemeinschaft teilzunehmen.

## Abstract (en)

**Background:**

In the second half of 2023 the Senate for Science, Health and Nursing of Berlin commissioned an explicit gerontological expert report from the team of authors, which was intended to provide suggestions for the modernization and concretization of § 71 of the German Social Code Act (SGB) XII, i.e., the central social law basis for so-called care of older people. It was completed by the end of 2023. The objective of the report was to provide suggestions for a Berlin Assistance for Old People Structural Law that considers the differentiation and diversity of the life phase of old age by identifying, analyzing and describing the necessary individual services.

**Aim of the article:**

The approach and the central results of the report are presented and discussed.

**Material and methods:**

In a multistep procedure, combined with a multimodal research approach, various methods were combined in order to generate a comprehensive understanding of the current situation of so-called assistance for older people and gerontological justifications for its services. Critical and selective literature reviews, document analysis and qualitative guideline-based expert interviews were used.

**Results and discussion:**

The expert report contains concrete recommendations for an age phase and life situation-orientated development of individual services according to § 71 SGB XII. This relates to approaches, forms and topics of counselling that enable and support successful ageing for all older people. The report also explicitly identifies objects of necessary cash and non-cash benefits, which, even in precarious situations in old age, aim to strengthen the ability for self-help and provide the opportunity to participate independently in community life.

## Hintergrund

Altenhilfe ist gesetzlich im § 71 SGB XII verankert und als eine Aufgabe im Bereich der kommunalen Daseinsvorsorge einzuordnen, wenngleich hierfür ein breiter Ermessensspielraum besteht [1]. Die sog. Altenhilfe soll dazu beitragen, Schwierigkeiten, die in der Lebensphase Alter entstehen können, zu verhüten, zu überwinden oder zu mildern und alten Menschen die Möglichkeit bieten, selbstbestimmt am Leben in der Gemeinschaft teilzunehmen und ihre Fähigkeit zur Selbsthilfe zu stärken. Neben diesen Zielen werden einzelne Leistungen der sog. Altenhilfe exemplarisch im § 71 Abs. 2 SGB XII aufgeführt. Allerdings geben die weit gefassten Formulierungen keine eindeutige rechtliche Orientierung für die vielfältigen Bedarfe und Aufgaben der Sozialen Altenarbeit – vielmehr bleiben sie in Bezug auf konkrete Leistungen unscharf und sehr vage. Vor allem wird in der gegenwärtigen Form der Differenziertheit und Diversität von Lebenslagen und -entwürfen alter Menschen zu wenig Rechnung getragen. Und es werden bislang *„so relevante Bereiche wie Prävention und Bildung nur unzureichend gesetzlich abgesichert“* [3].

Wenn die Kommunen jedoch dafür zu sorgen haben, wie in einem jüngeren Rechtsgutachten betont wird, *„dass die Vorkehrungen und Einrichtungen gegeben sind, die nötig sind, damit die altenhilferechtlichen Leistungen jedenfalls auf einem Mindeststandard wirksam erbracht werden können“* [6], stehen sie letztlich vor der Frage, welche Leistungen, die im oben genannten Verständnis als sinnvoll und notwendig eingeordnet werden, vorgehalten werden müssen.

Dazu ist grundlegend anzumerken, dass es für die Bearbeitung sozialer Fragen und Bedarfe in der länger werdenden Lebensphase Alter noch immer keine einheitliche gesetzliche Grundlage gibt, die mit dem SGB VIII, also der Kinder- und Jugendhilfe, vergleichbar wäre. Vielmehr sind die rechtlichen und finanziellen Ansprüche alter Menschen insgesamt unterschiedlichen Sozialgesetzbüchern (SGB V, SGB VI, SGB IX, SGB XI, SGB XII) und Verwaltungsvorschriften zugeordnet, damit stark zersplittert und ohne einheitliche Logik.

Diese Erkenntnis ist nicht neu und hat in einschlägigen gerontologischen Fachdebatten immer wieder zur Forderung nach einem einheitlichen Altenhilfestrukturgesetz geführt, bisher allerdings ohne erkennbaren Fortschritt [2]. Solange § 71 SGB XII bundesgesetzlich nicht reformiert wird, können nur landesgesetzliche Regelungen die Leistungen der Altenhilfe für die Kommunen gegenständlicher fassen und damit zur sog. Gleichwertigkeit der Lebensverhältnisse im Alter beitragen. Als erstes Bundesland stellt sich nun Berlin dieser Aufgabe. Angestoßen wurde dieser Prozess durch den dortigen Landesseniorenbeirat, der 2019 beschlossen hat, ein Berliner Altenhilfestrukturgesetz auf den Weg zu bringen. Diese Zielsetzung wurde in der Koalitionsvereinbarung des aktuellen Berliner Senats aufgegriffen. Für die Entwicklung eines Gesetzesvorschlags wurde vom Landesseniorenbeirat ein Dialogprozess initiiert, an dem eine Redaktionsgruppe aus Expert*innen und eine Steuerungsgruppe (unter Beteiligung der für die Altenhilfe zuständigen Senatsverwaltung und der im Berliner Abgeordnetenhaus vertretenen Fraktionen) beteiligt waren [7]. Vor diesem Hintergrund hat das Land Berlin das gerontologische Gutachten in Auftrag gegeben; dieses steht im Fokus des vorliegenden Beitrags.

Das erstellte gerontologische Gutachten zu fachlich begründeten Einzelleistungen nach § 71 SGB XII soll folglich mit dazu beitragen, die offenkundigen Unschärfen zu beseitigen. Es bildet ein bedeutsames Element für die Ausgestaltung eines Berliner Altenhilfestrukturgesetzes auf Grundlage des § 71 SGB XII. Explizit als gerontologisch fundiertes Gutachten gefordert und zu verstehen, ordnet es die relevanten Fragen in die aktuellen Fachdiskurse der Gerontologie, mit dem Schwerpunkt auf der fachlichen Orientierungen der Sozialen Gerontologie, ein.

## Zentrale Zielsetzungen des Gutachtens

Im betreffenden Gutachten ging es um die Beschreibung möglicher Einzelleistungen, die durch einschlägige Literaturrecherchen und auf Empirie gestützt, konkrete Zugänge und Gegenstände darlegen sollten. Zentral wurde die Frage beleuchtet, was es braucht, um Leistungen der sog. Altenhilfe „neu zu denken“. Die damit verbundenen Aufgabenstellungen lassen sich in folgende drei Bereiche bündeln:

- Adressat*innen: Kategorisierung von Personengruppen mit Bedarf an Einzelleistungen nach § 71 SGB XII, inklusive einer Abschätzung möglicher Alterskategorien und -grenzen.
- *Einzelleistungen:* Analyse und Beschreibung von für die Entwicklung von Einzelleistungen nach § 71 SGB XII zu berücksichtigenden Kriterien und Parameter, die den aktuellen Kenntnisstand sowie neuere Entwicklungen und diverse Bedarfslagen verschiedener Adressat*innengruppen im Alter umfassen und damit auch über die bislang in § 71 Abs. 2 SGB XII benannten Leistungen hinausgehen.
- *Berliner Bedarfe:* Beschreibung der aktuellen Situation, absehbar künftiger Entwicklungen von Bedarfen sowie der ungefähren Zahl der Personen mit potenziellem Anspruch auf Leistungen nach § 71 SGB XII in den Berliner Bezirken.

## Theoretische und methodische Zugänge

Zur Bearbeitung der skizzierten Aufgaben des Gutachtens bedurfte es gerontologischer Grundlagen, auf deren Basis die Ausdifferenzierungen zur Lebensphase Alter begründet werden können. Hierzu war es aus Sicht der Gutachter*innen nicht ausreichend, die in der Gerontologie bekannten Diskussionszusammenhänge zu Altersdefinitionen und -kategorien deskriptiv wiederzugeben. Vielmehr sollten diese auch kritisch für die Zielsetzungen des vorliegenden Gutachtens in einen Fachdiskurs eingeordnet werden. Daher wurde ein *kritisches Literaturreview* gewählt, das die vorhandene Literatur selektiv und kritisch, mit Blick auf Kontroversen und Lücken analysiert. Im Gegensatz zu einer systematischen Übersichtsarbeit hat dieses Vorgehen jedoch nicht den Anspruch, den gesamten Literaturstand repräsentativ zu erfassen und unter Bewertung der Studienqualität auszuwerten [13]. Dafür wurden spezifische gerontologische ebenso wie allgemeine sozialwissenschaftliche Datenbanken genutzt und die Ergebnisse durch eine händische Recherche ergänzt.

Darauf aufbauend erfolgte ein *selektives Literaturreview*, das altersspezifische Ausprägungen von Lebenslagen und sozialer Ungleichheit fokussiert, um diverse Bedarfslagen von älteren Adressat*innen zu erfassen und damit nicht nur die Personengruppen differenzierter zu umschreiben, sondern zugleich Hinweise auf Kriterien und Parameter zu Einzelleistungen aus gerontologischer Perspektive generieren zu können. Dieses selektive Literaturreview wurde mit dem Ziel durchgeführt, vergleichsweise kurzfristig einen breiten Überblick in Bezug auf den Literatur- und Forschungsstand zu diesen Themenkomplexen zu gewinnen [15]. Zur Sicherung der methodischen Qualität wurden bei dieser Recherche die typischen Berichtselemente eines systematischen Literaturreviews genutzt, indem das Vorgehen anhand eines Flussdiagramms gemäß den „Preferred Reporting Items for Systematic Reviews and Meta-Analyses“ (PRISMA) dokumentiert wurde (Ein- und Ausschlusskriterien, Datenbanken und Suchverlauf, Screening und Prüfung auf Eignung).

Um jüngere Entwicklungen und spezifische Bedarfslagen von Adressat*innengruppen im Alter auch qualitativ auf Basis von aktuellem Spezialwissen berücksichtigen zu können, wurden zudem gezielt die ergänzenden Einschätzungen von drei Expert*innen erhoben: zu älteren Adressat*innengruppen, denen in der Literatur ein Bedeutungszuwachs zugeschrieben wird („lesbian, gay, bisexual, transsexual/transgender, intersexual, queer [LGBTIQ*] communities“ und Menschen mit Migrationsgeschichte) sowie zu Rahmenbedingungen, die das Alter zunehmend prägen (Technikentwicklung und Digitalisierung).

Für die weitergehende Identifizierung und Einordnung von Einzelleistungen der sog. Altenhilfe erfolgten auf Grundlage bislang vorhandener kommunaler Regelungen Dokumentenanalysen sowie weitere Expert*inneninterviews. Da die in § 71 Abs. 2 Nrn. 1–6 SGB XII aufgeführten Leistungen der Altenhilfe zwar gewisse Konkretisierungen vornehmen, aber inhaltlich immer noch sehr offen beschrieben sind und eine *„beispielhafte, nicht abschließende Aufzählung“* [1] darstellen, bestand das zentrale Ziel darin, konkrete Beispiele aus bereits existierenden Regelungen zu Einzelleistungen sowie Erfahrungen in der konzeptionellen Entwicklung und praktischen Gewährung zu erheben.

Die *qualitative Dokumentenanalyse* [5] erfolgte auf Basis einer schlagwortgebundenen Internetrecherche zur Identifizierung und zur Auswahl von Regelungen zum § 71 SGB XII auf kommunaler Ebene. Der Recherchefokus lag auf ausdifferenzierten Regelungen, die Einzelleistungen näher beschreiben. Im zweiten Schritt wurde stichprobenartig in Bezug auf ausgewählte größere Kommunen recherchiert, welche Regelungen dort zur Altenhilfe im Internet dokumentiert sind. Die identifizierten Internetseiten und Dokumente wurden hinsichtlich der Einzelleistungen inhaltsanalytisch induktiv ausgewertet.

Die Expert*inneninterviews adressierten im Sampling Personen in leitenden Funktionen der kommunalen Verwaltung aus Behörden für Soziales/Sozialämtern und Fachbereichen/Abteilungen der Altenhilfe (im weiten Sinne), die – in Anlehnung an Meuser und Nagel [12] – „Kontextwissen“ zu Leistungen nach § 71 SGB XII sowie „Betriebswissen“ zu Hintergründen, Inhalten und Praxen in Bezug auf den Leistungskatalog der eigenen Kommune besitzen. Die Auswahl erfolgte im Anschluss an die Dokumentenanalyse einerseits nach Kommunen, die einen für das Gutachten interessierenden Leistungskatalog aufweisen, sodass damit insbesondere Zugänge zu Betriebswissen über bestehenden Regelungen geschaffen wurden. Andererseits wurden Personen gewählt, die (auch) über Kontextwissen zu Leistungen nach § 71 SGB XII verfügen, da sie nicht nur leitend in kommunalen Abteilungen zur Altenhilfe, sondern auch an überregionalen Gremien und Fachveranstaltungen im Bereich der Altenhilfe mitwirken. Ferner wurde ein Interview geführt mit einer*m juristischen Expert*in aus der Wissenschaft, der*die über juristisches Kontextwissen zum § 71 SGB XII verfügt und an der Entwicklung des Berliner Gesetzesentwurfes beteiligt war.

Die Interviews (*n* = 5) wurden vollständig, in leicht geglätteter Sprache transkribiert und im Sinne der inhaltlich strukturierenden Inhaltsanalyse [10] ausgewertet.

**Abb. 1:**
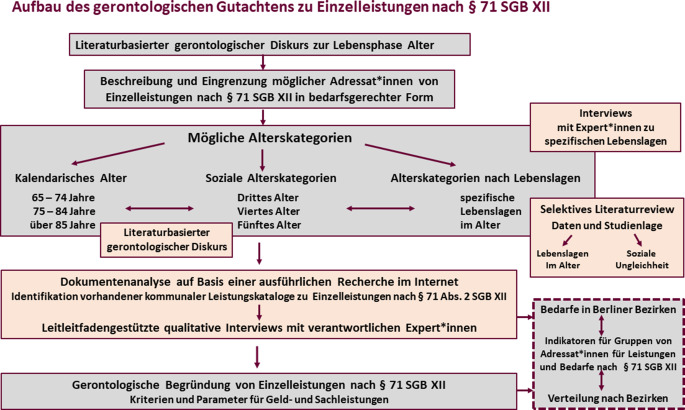
Aufbau des gerontologischen Gutachtens zu Einzelleistungen nach § 71 SGB XII. (Aus Engler et al. [4, S. 9])

Das gesamte methodische Vorgehen sowie dessen schrittweiser Aufbau und inhärente Logik ergeben sich aus der Übersicht in Abb. 1.

## Ergebnisse und deren Diskussion

Als Ergebnis des *kritischen Literaturreviews* in Bezug auf mögliche *Kategorisierungen von Personengruppen* konnte die Problematik differenziert ausgeführt werden, dass Alterskategorisierungen stets kontextabhängig sind und – je nach Bezugsgröße – mit unterschiedlichen Schlussfolgerungen verbunden werden können und müssen [14]. Insgesamt besteht Einigkeit darüber, dass sich Personen im gleichen kalendarischen Alter in Abhängigkeit von individuellen Dispositionen und Umweltbedingungen erheblich unterscheiden können [14], und dass biologische, psychische oder soziale Dimensionen nicht in unmittelbare Beziehung zum kalendarischen Alter gesetzt werden können. Soziale Alterskategorien können in diesem Kontext aber eine Orientierung bieten, die sich über alterstypische Lebensereignisse sowie Entwicklungsaufgaben definieren und auch Hinweise auf mögliche Beratungsthemen und -anliegen in unterschiedlichen Altersphasen anbieten (s. Beitrag 3 dieses Themenschwerpunkts [9]). Die Systematik der sog. Sozialen Alterskategorien versteht sich, unabhängig vom kalendarischen Alter, als eine neue Periodisierung des Lebenslaufs, orientiert an den sozialen Bedingungen. Dieser veränderte Blick auf das Alter ist verbunden mit sich ausdifferenzierenden Perspektiven auf biografische Prägungen sowie auf daraus entstehende Lebensmuster und Lebenslagen [8].

Auch wenn das kalendarische Alter als (alleiniger) Indikator für eine Adressierung durch die sog. Altenhilfe im Gutachten als unzureichend markiert wurde, kann dieses nicht in Gänze ausgeblendet werden. Denn für das Verwaltungshandeln in Bezug auf den § 71 SGB XII haben Orientierungen an Altersgrenzen durchaus Relevanz. Deshalb waren im Rahmen des Gutachtens zwangsläufig auch kalendarische Altersdimensionen erforderlich, da Abschätzungen von Altersgrenzen und Quantifizierungen der Personengruppen für die Berliner Bezirke explizit gewünscht waren. Als kalendarische Altersgrenzen, verbunden mit den genannten Einschränkungen, wurde deshalb im Gutachten unterschieden zwischen Personen im Alter von 65 bis 74 Jahren, einer Kategorie der 75- bis 84-Jährigen und einer dritten Gruppe der Menschen ab 85 Jahren – auch weil sich relevante Studien an dieser Unterscheidung orientieren.

Im zentralen Ergebnis des *selektiven Literaturreviews in Bezug auf Lebenslagen und soziale Ungleichheiten* in der Lebensphase Alter wird deutlich erkennbar, dass Einschränkungen bzw. Benachteiligungen ebenso wie Ressourcen häufig mit den spezifischen Lebenslagen im Prozess des Alterns zusammenhängen. In ihren Ausprägungen sind sie aber wiederum sehr heterogen, vielschichtig und nicht monokausal zu denken. Zudem sind sie immer eng verwoben mit vorherigen Lebensabschnitten und auch den Handlungsspielräumen, die sich dort zeigen und die dort genutzt werden konnten – oder aufgrund sozialstruktureller Benachteiligungen eben auch nicht [11].

In Bezug auf die Frage nach Kriterien für die Entwicklung von Einzelleistungen nach § 71 SGB XII ist aber auch festzustellen, dass die Lebenslagen spezifischer Adressat*innengruppen (z. B. der LGBTIQ Community) inhaltlich nicht zu besonderen Dimensionen an Bedarfen führen. Diese sind vielmehr inhaltlich jeweils identifizierbar in den Handlungsspielräumen, die sich beispielsweise auf Beratungen zur finanziellen Situation, zur Wohnsituation, zu Angeboten der sozialen Teilhabe sowie Bildung und Kultur, Möglichkeiten der Beteiligung oder Unterstützungsleistungen im Vor- und im Umfeld von Pflege beziehen können. In der Beratung zu Angeboten und Leistungen der sog. Altenhilfe ist allerdings zu beachten, dass die Lebenslagen spezifischer Adressat*innengruppen in diesen inhaltlichen Bereichen zu unterschiedlichen Voraussetzungen und Bedingungen für individuelle Bedarfe und Ressourcen führen. Damit ist auch zu bedenken, dass verschiedene Merkmale der Differenz v. a. strukturell zu sozialer Ungleichheit, die in der individuellen Analyse der Bedarfslage zu berücksichtigen sind, führen. Insgesamt geht es bei der Feststellung von Bedarfen im Sinne des § 71 SGB XII also darum, diversitätssensibel zu reagieren und das Verwaltungshandeln danach auszurichten. Und genau dafür müssen die zuständigen Mitarbeiter*innen in den Verwaltungen entsprechend geschult werden.

Zusammenfassend ist als Ergebnis der *Internetrecherche und Dokumentenanalyse zum aktuellen Stand kommunaler Regelungen* festzuhalten, dass die Mehrzahl der Kommunen lediglich über allgemein gehaltene Hinweise auf Leistungen der Altenhilfe nach § 71 SGB XII, die in unterschiedlichen Varianten etwa Ziele und/oder Leistungen der Altenhilfe benennen, verfügt. Nur bei einer Minderheit von Kommunen existieren konkrete Richtlinien, Fachanweisungen bzw. Arbeitshilfen und Leistungskataloge zum § 71 SGB XII. Nähere Hinweise auf Ansätze und Formen der Beratung im Rahmen der Altenhilfe sind hierbei wiederum nur vereinzelt öffentlich zu finden und beziehen sich auf aufsuchende Beratungsformen, die einerseits insbesondere als Hausbesuch, auch präventiv, sowie andererseits in digitaler Form durchgeführt werden können. An die Beratung anschließende Ansätze reichen von der Kontaktvermittlung über – bei komplexen Beratungssituationen – Case Management bis hin zur Krisenintervention. Es ist dabei davon auszugehen, dass Ausgangspunkt stets ein beratender Erstkontakt ist. Konkrete Hinweise auf mögliche Geld- und Sachleistungen nach § 71 Abs. 2 SGB XII liegen ebenfalls nur bei einzelnen Kommunen, die über eine Art Leistungskatalog hierzu verfügen, vor. Auch wenn manche Einzelleistungen in verschiedenen Leistungskatalogen analog zu finden, bleiben diese in Inhalt und Umfang der Leistungen durchaus heterogen.

Die entsprechenden Expert*inneninterviews mit Repräsentant*innen der kommunalen Ebene haben aber auch deutlich gemacht, dass die Auseinandersetzung mit dem § 71 SGB XII in der kommunalen Politik und Verwaltung in den letzten Jahren an Bedeutung gewonnen hat. Übergeordneter Anlass zur Entwicklung bzw. zur Planung einer kommunalen Regelung zum § 71 SGB XII ist der deutlicher werdende Bedarf, auf die mit der demografischen Alterung verbundenen Aufgaben konkret auf kommunaler Ebene zu reagieren. Die Entwicklung entsprechender Regelungen zum § 71 SGB XII, insbesondere von den hier für Einzelleistungen relevanten Leistungskatalogen, erfolgt v. a. auf Basis von Erfahrungen in der Beratungspraxis der Altenhilfe und Gewährungspraxis zu Geld- und Sachleistungen sowie über die Abgrenzung zu anderen Sozialleistungen und die Orientierung an den Regelungen anderer Kommunen. Von zentralem Interesse für das Gutachten war auch, dass noch kein einheitliches bzw. zumindest analoges Verständnis zum Begriff der Einzelleistungen zu existieren scheint. Zugleich erscheint unklar, was neben der Beratung unter dem in § 71 Abs. 2 SGB XII mehrmals damit verbunden benannten Aspekt der „Unterstützung“ gemeint ist. In den Kommunen existieren ferner unterschiedliche Altersgrenzen, die der Orientierung dienen, bei entsprechenden Bedarfen aber wiederum in Einzelfallentscheidungen unterschritten werden können.

**Abb. 2:**
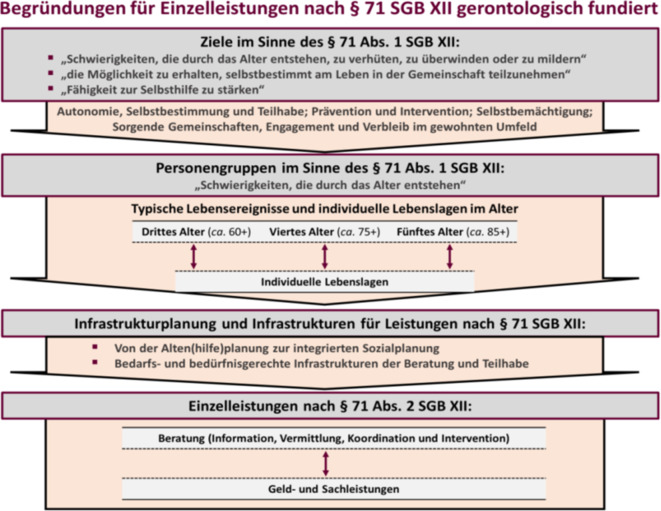
Gerontologisch fundierte Begründung für Einzelleistungen nach § 71 SGB XII. (Aus Engler et al. [4, S. 180])

Auf Grundlage der skizzierten Analyseergebnisse erfolgte im Rahmen des Gutachtens eine zusammenführende gerontologische Begründung von Einzelleistungen gemäß § 71 SGB XII. Diese wurde eingeordnet in den Gesamtkontext der sog. Altenhilfe gemäß § 71 SGB XII, um auch auf wesentliche Voraussetzungen zur Entwicklung und zur Umsetzung von Einzelleistungen hinzuweisen. Dem Aufbau der Abb. 2 folgend wurden dafür zunächst Ziele, Personengruppen und Infrastrukturen für Einzelleistungen ausgehend von Inhalten des § 71 SGB XII – wiederum aus gerontologischer Sicht – skizziert, um dann relevante Einzelleistungen im Sinne des § 71 SGB XII näher auszuführen und zu begründen.

Im letzten Schritt wurden auf Basis dieser gerontologischen Zugänge und Begründungen in einer Übersichtsmatrix erkennbar notwendige Einzelleistungen als mögliche Themen der Beratung sowie gleichzeitig als Gegenstände von Geld- und Sachleistungen dargestellt (Tab. 1).

**Tab. 1:** Beratungsthemen sowie Geld- und Sachleistungen gemäß § 71 SGB XII, differenziert nach Alterskategorien und Lebenslagen

Soziale Alterskategorien/kalendarisches Alter	Lebenslagen						
	Vermögens- und Einkommensspielraum	Materieller Versorgungsspielraum	Kontakt‑, Kooperations- und Aktivitätsspielraum	Lern- und Erfahrungsspielraum	Dispositions- und Partizipationsspielraum	Regenerationsspielraum zur Gesunderhaltung	Unterstützungsspielraum
	*Ökonomische Situation der Individuen, v.* *a. ihr Einkommen aus Beschäftigungsverhältnissen, Renten, Einkommen aus Vermögen, Transferzahlungen*	*Wohnbedingungen (Wohnort, Wohnungsart, Wohnungsgröße, Ausstattung der Wohnung)*	*Möglichkeiten zu Aufnahme und Aufrechterhaltung sozialer Kontakte (beruflich, privat)*	*Möglichkeiten zu persönlicher Weiterentwicklung, Entfaltung und Gestaltung von spezifischen Interessen, Ausmaß räumlicher und sozialer Mobilität und von Wohn- und Umweltbedingungen*	*Chancen und Möglichkeiten der politischen und gesellschaftlichen Mitwirkung als Voraussetzung zu Mitgestaltung und Übernahme von Verantwortung durch die Gesellschaft*	*Erholung, körperliche und geistige Regeneration und Erhalt der eigenen Gesundheit*	*Verfügbare private und öffentliche Ressourcen – pflegende An- und Zugehörige – ambulante, teilstationäre, stationäre und offene Angebote der Pflege und Unterstützung*
*3. Alter* Veränderung familiärer Situation Ausscheiden aus dem Erwerbsleben Veränderungen in sozialen Netzwerken Mehr freie Zeit zur sinnvollen Gestaltung Neuorientierung (neue Rollen und Aufgaben)	*Beratung zu den Themen*						
	Übergang in die nachberufliche Phase und finanzielle Situation^(3)^ Zusätzliche finanzielle Ressourcen neben der Rente, z. B. geringfügige Beschäftigung, Übungsleiterpauschale* Grundsicherung	Wohnungsanpassung^2^ Frühzeitiger Umzug in seniorengerechte Wohnform^2^ Umzugshilfen^2^	Angebote zur sozialen Teilhabe – auch generationenverbindend^5^ Strukturen zur Vernetzung mit ähnlich orientierten Personengruppen^5^ Aufbau neuer sozialer Netzwerke^5^ Digitale Vernetzung Personennahverkehr^5^	Bildungs- und Kulturangebote^5^ Neuorientierung in der nachberuflichen Phase^(3)^ und Aktivitäten zur Gestaltung der freien Zeit^5^	Ehrenamt/Engagement^1^ Vereinsarbeit^1^ Politische Partizipation durch Mitarbeit in Gremien, wie Seniorenbeirat, Quartiersrat etc.^1^	Bewegungs- und Sportangebote^5^ Angebote zu Gesundheitsförderung bzw. Krankheitsprävention* Psychosoziale Begleitungsangebote und Selbsthilfegruppen*	Soziale, medizinische und pflegerische Dienstleistungen^3, 4^ Entlastungs- und Unterstützungsangebote für pflegende An- und Zugehörige^3,4^
	*Geld- und Sachleistungen*						
	Siehe in weiteren Lebenslagendimensionen	Zuschüsse zu baulichen Maßnahmen und Erhaltungsaufwendungen (z. B. Abbau von Barrieren, rutschfester Bodenbelag)^2^ Umzugsbedingte Aufwendungen (z. B. Auf- und Abbau von Möbeln, Elektroanschlüsse)^2^	Fahrtkostenzuschuss (z. B. zu Fahrmarken)^5^ Reisebeihilfe (Fahrt zu Familienangehörigen)^5^ Zuschuss zu Kosten der analogen und digitalen Vernetzung (z. B. Telefon, Internet)^5^	„Kulturpass“^5^ Kostenübernahme von Eintrittskarten für Konzert, Museum, Kino, Theater etc. (begrenzte Anzahl/Jahr)^5^ Bildungsgutscheine^5^	Zuschuss oder Übernahme von Mitgliedsbeitrag für Vereine^1^ Übernahme von Fahrtkosten^1^	Mitgliedsbeitrag eines Sportvereins etc.^5^ „Gesundheitspass“*	Finanzierung von Hintergrunddiensten (z. B. Schlüsselaufbewahrung für Notfälle) ^2^ Befreiung oder Reduzierung von finanziellen Eigenanteilen^3^
*4. Alter* Ausdünnung sozialer Netzwerke Erfahren beginnender physischer, psychischer und/oder kognitiver Einschränkungen (persönlich und im Umfeld) Kritische Überprüfung der Wohnsituation Bewältigung typischer kritischer Lebensereignisse (Verluste von Personen und Ressourcen)	*Beratung zu den Themen*						
	Grundsicherung	Verbesserung der Wohnbedingungen, v. a. bei allmählicher Einschränkung der Mobilität^2^ Alternativen zur aktuellen Wohnsituation^2^	Angebote für soziale Teilhabe im Nahraum^5,6^ Digitale Vernetzung im Quartier^5,6^ Nutzung von Apps und digitalen Plattformen^5,6^ Fahrdienste^5,6^ Senior*innenreisen^5^	Passende Bildungs- und Kulturangebote^5^ Angebote der Begegnung im Nahraum^5^	Ehrenamt/Engagement^1^ Politische Partizipation durch Mitarbeit in Gremien – auch online^1^	Angepasste Sportangebote^5^ Angebote zur Gesundheitsförderung* Öffentliche Mittagstischangebote (gesunde Ernährung in Gemeinschaft)*	Soziale, medizinische und pflegerische Dienstleistungen^3,4^ Hilfe und Unterstützung im Alltag^3,4^ Entlastungs- und Unterstützungsangebote für pflegende An- und Zugehörige^3,4^
	*Geld- und Sachleistungen*						
	Siehe in weiteren Lebenslagendimensionen	Finanzielle Umbau- oder Umzugshilfen (s. 3. Alter)^2^ Einmaliger Zuschuss zu alltagsrelevanten Haushaltsgegenständen (wie Mikrowelle oder Waschmaschine)*	Kostenübernahme für analoge und digitale Vernetzung (z. B. Telefon, Internet und WLAN)^6^ Zuschüsse für Fahrdienste (z. B. Nachttaxi)^6^ Kostenzuschuss oder -übernahme zu Senior*innenfahrten oder Kurzfreizeiten^5^	„Kulturpass“^5^ Kostenübernahme von Eintrittskarten (s. 3. Alter)^5^ Bildungsgutscheine^5^	Kostenübernahme für Internet und WLAN^1^ Übernahme von Fahrtkosten^1^	Mitgliedsbeitrag eines Sportvereins etc. ^5^ „Gesundheitspass“ *	Finanzierung von Hintergrunddiensten (s. 3. Alter)^3,4^ Hausnotruf^3^ Hauswirtschaftliche Dienste – ohne Pflegegrad (z. B. Einkaufsdienste)^3,4^ Leistungen körperbezogener Pflege unterhalb von Pflegegrad 2 (z. B. Hand- und Fußpflege)^3,4^ Kostenzuschuss für Übernahme allgemeiner Mieter*innenpflichten (z. B. Reinigung von Treppenhaus, Winterdienst, Gartenpflege)^2^
*5. Alter* Erleben von Autonomieverlust und Einschränkungen der persönlichen Reichweite sowie sozialer Kontakte Bedarf an Unterstützung, Hilfe und Pflege Auseinandersetzung mit eigener Endlichkeit Verstärkte Singularisierung (soziale Isolation)	*Beratung zu den Themen*						
	Hilfe zur Pflege	Pflegeadäquates Wohnumfeld^2,3^ Zuschüsse zur Wohnraumanpassung ^2,3^ Smart Home^3^	Nachbarschaftliche Hilfen und Unterstützung^5,6^ Besuchsdienste^6^ Digitale Kommunikation^5,6^ Begleit‑, Fahr- und Mobilitätsdienste^5,6^	Zugehende Formen von Lernbegleitung^5^ Besuchsdienste^5^ Digitale Kommunikation^5^	Online-Beteiligung an partizipativen Prozessen^1^ Digitale Kommunikation^1^	Aktivierende Hausbesuche* Angebote geriatrischer Rehabilitation*	Pflegeberatung^3,4^ Diverse Versorgungssettings^3,4^ Entlastungs- und Unterstützungsangebote für pflegende An- und Zugehörige^3,4^
	*Geld- und Sachleistungen*						
	Siehe in weiteren Lebenslagendimensionen	Finanzielle Umbauhilfen^2^ Kostenübernahme für technische Assistenzsysteme^2,3^ Kostenübernahme für digitale Unterstützung diverser Pflegesettings^2,3^	Kostenübernahme für evtl. Eigenbeteiligungen^6^ Finanzielle Hilfen zur Nutzung digitaler Technik (z. B. Tablet)^6^ Zuschüsse für Begleit‑, Fahr- und Mobilitätsdienste^6^	Kostenübernahme für evtl. Eigenbeteiligungen^5^ Finanzielle Hilfen zu technischer und digitaler Ausstattung der Information (z. B. Radio, Fernsehgerät mit größeren Tasten, Tablet oder Laptop)^5^	Kostenübernahme für Internet und WLAN^1^ Finanzielle Unterstützung für Begleit‑, Fahr- und Fahrdienste^1^ Finanzielle Hilfen zur Nutzung digitaler Technik^1^	Kostenübernahme für evtl. Eigenbeteiligungen* Finanzielle Unterstützung für notwendige Fahrdienste^5^	Kostenübernahme zur Abnahme allgemeiner Mieter*innenpflichten (z. B. Treppenhausreinigung, Gartenpflege, Winterdienst) ^2^ Kostenübernahme für evtl. Eigenbeteiligungen bei SGB-XI-Leistungen^3^ Finanzielle Unterstützung für notwendige Fahrdienste^3^

§ 71 Abs. 2 SGB XII: Als Leistungen der Altenhilfe kommen insbesondere in Betracht:

^1^ Nr. 1: Leistungen zu einer Betätigung und zum gesellschaftlichen Engagement, wenn sie vom alten Menschen gewünscht wird

^2^ Nr. 2: Leistungen bei der Beschaffung und zur Erhaltung einer Wohnung, die den Bedürfnissen des alten Menschen entspricht

^3^ Nr. 3: Beratung und Unterstützung im Vor- und im Umfeld von Pflege, insbesondere in allen Fragen des Angebots an Wohnformen bei Unterstützungs‑, Betreuungs- oder Pflegebedarf sowie an Diensten, die Betreuung oder Pflege leisten

^4^ Nr. 4: Beratung und Unterstützung in allen Fragen der Inanspruchnahme altersgerechter Dienste

^5^ Nr. 5: Leistungen zum Besuch von Veranstaltungen oder Einrichtungen, die der Geselligkeit, der Unterhaltung, der Bildung oder den kulturellen Bedürfnissen alter Menschen dienen

^6^ Nr. 6: Leistungen, die alten Menschen die Verbindung mit nahestehenden Personen ermöglichen

* Sonstige, bislang nichtdefinierte Leistungen

^(3)^ Abs. 3: Leistungen nach Absatz (1) sollen auch erbracht werden, wenn sie der Vorbereitung auf das Alter dienen

Abschließend sei in Adressierung der Politik hervorzuheben, dass es wünschenswert wäre, wenn andere Bundesländer dem Vorbild von Berlin auf dem Weg zu einem Altenhilfestrukturgesetz folgen, um letztlich eine Umsetzung des vagen § 71 SGB XII im Sinne einer konsistenten und konsequenten Förderung der Autonomie, Selbstbestimmung und Teilhabe von älteren und alten Menschen zu gewährleisten.

## Fazit für die Praxis

Altenhilfe neu denken, bedeutet im Sinne des skizzierten Gutachtens:

- Einzelleistungen nach §  1 SGB XII nicht nur als Vermittlung einkommens- und vermögensabhängiger Geld- und Sachleistungen in den Blick zu nehmen, sondern diesen auch verstärkt auf die einkommensunabhängige Beratung zu Angeboten und Leistungen – inklusive damit verbundener Formen der diversitätssensiblen Information, Vermittlung, Koordination und Intervention – zu weiten. Hier sind insbesondere Fachkräfte der Profession Soziale Arbeit mit ihrem Kompetenzprofil gefragt.
- Die Einbettung von Angeboten und Leistungen in entsprechende Infrastrukturen sowie deren bedarfsgerechte und partizipative Planung.
- Die Etablierung einer neuen Betrachtungslogik, Leistungen der sog. Altenhilfe, die in § 71 Abs. 1 SGB XII als „Schwierigkeiten, die durch das Alter entstehen“ charakterisiert werden, einerseits gerontologisch zu begründen und andererseits im Sinne von Befähigung auf die Ermöglichung Sozialer Teilhabe zu orientieren.
- Statt fixierter Altersgrenzen eignen sich Soziale Alterskategorien besser zur Bewertung des individuellen Bedarfs. Ausgehend von jeweils alterstypischen Lebensereignissen und Entwicklungsaufgaben sowie dazu quer liegenden Lebenslagendimensionen lassen sich die Leistungen nach § 71 SGB XII gerontologisch fundiert begründen.
