# Erratum zu: Gerontologisches Gutachten zu fachlich begründeten Einzelleistungen nach § 71 SGB XII

**DOI:** 10.1007/s00391-024-02376-3

**Published:** 2024-10-11

**Authors:** Stefanie Engler, Christian Bleck, Cornelia Kricheldorff

**Affiliations:** 1 Evangelische Hochschule Freiburg, Bugginger Straße 38, 79114 Freiburg, Deutschland; 2 Hochschule Düsseldorf, Düsseldorf, Deutschland; 3 Katholische Hochschule Freiburg, Freiburg, Deutschland

**Erratum zu:**

**Z Gerontol Geriat 2024**

10.1007/s00391-024-02348-7

In diesem Artikel hätte der Satz „Dieses zentrale Ergebnis ist ausführlich im Zusatzmaterial online nachvollziehbar, während der vorliegende Beitrag selbst die wissenschaftliche Fundierung und den Begründungweg des gerontologischen Gutachtens für das Land Berlin erörtert hat.“ gelöscht werden sollen.

Der Originalbeitrag wurde korrigiert.

